# Supplementary material for: One-carbon fixation via the synthetic reductive glycine pathway exceeds yield of the Calvin cycle
Source: Nat Microbiol. 2025 Feb 27;10(3):646–53. doi: 10.1038/s41564-025-01941-9 (PMC11879842; doi:10.1038/s41564-025-01941-9)
Supplement: Supplementary file 1 — Supplementary Information [file 41564_2025_1941_MOESM1_ESM.pdf]

nature microbiology

Supplementary information https://doi.org/10.1038/s41564-025-01941-9

One-carbon fixation via the synthetic reductive glycine pathway exceeds yield of the Calvin cycle

In the format provided by the authors and unedited

Supplementary Information

Tables:

Supplementary Table 1 | Mutations of CRG5.5 compared to CRG4.5. Chromosome is abbreviated as Chr.

Chr.	position	mutation	annotation	gene	description
1	1,499,624	Tn5-P_{14}-C1 insertion	Insertion 44 nt upstream of start ATG	*phaP1*	phasin (PHA-granule associated protein)
1	2,072,499	Tn5-P_{14}-C1 insertion	Insertion after I175 (525/2613 nt)	*acnM*	methyl-cis-aconitic acid hydratase/Aconitate hydratase
2	222,178	(AGGCCG CGCGGC) 1→2	coding (1489/3063 nt)	*sbcC*	DNA repair exonuclease, SbcC
2	926,039	G→C	L32V (CTC→GTC)	*B0818*	transcriptional regulator, XRE-family
pC3	1	Δ5,923 bp			pSEVA331-PphaC1-sdaA-glyA

Supplementary Table 2 | Mutations of CRG6 Δ*dadA6* compared to CRG5.5. Chromosome is abbreviated as Chr.

Chr.	position	mutation	annotation	gene	description
1	268,712	Δ1 bp	coding (10/855 nt)	*A0258*	short chain dehydrogenase
1	3,461,712	+G	coding (823/969 nt)	*A3204*	transcriptional regulator, LysR-family
2	159,389	+G	Insertion 22 nt upstream of start ATG	*B0138*	transcriptional regulator, IclR-family/conserved hypothetical transmembrane protein
2	2,234,079	Tn5-P_3-C3 insertion	Insertion at (1279/1935)	*B1976*	outer membrane receptor, TonB dependent
2	571.876	1330 bp→5 bp		[dadA6]	[dadA6]

Supplementary Table 3 | Tn5 promoter C1 and C3 operon insertion sites of CRG5 and CRG6 clones.
*location on chromosome (Chr) 1 or 2 of the P_2-C1 insertion is unclear, as it inserted in a large duplicated region with identical sequence on chromosome 1 and 2.

Strain	Chr	Position (nt)	Insertion locus	Direction
CRG5 gP_{14}-C1	1	1,499,624	*phaP1/H16_A1382*	sense
	1	2,072,499	*acnM*	sense
CRG5 gP_{PhaC1}-C1	1	269,292	*h16_A0258*	sense
CRG5 gP_3-C1	1	1,499,668	*phaP1/H16_A1382*	sense
CRG5 gP_4-C1	1	1,499,668	*phaP1/H16_A1382*	sense
CRG5 gP_2-C1	1	3,942,813	*H16_A3661 *or H16_B0502*	anti-sense
CRG5 gP_{14}-C1-2	1	1,499,668	*phaP1/H16_A1382*	sense

CRG6 gP$_3$-C3	2	2,234,079	*H16_B1976*	sense
CRG6 gP$_3$-C3-2	1	2,070,024	*prpB*	sense

Supplementary Table 4 | Overview of formatotrophic and methylotrophic yields and growth rates.

Pathway	Organism	gCDW mol^{-1}	Dt (h)	μ h^{-1}	Substrate	C (M)	Ref.
rGlyP	*C. necator*	4.52	8.0	0.087	formate	0.08	This study
CBB	*C. necator*	4.056	3.8	0.18	formate	-	[1]
rGlyP	*E. coli*	2.3	8.0	0.087	formate	0.109	[2]
rGlyP	*C. necator*	2.6	12.0	0.058	formate	0.08	[3]
rGlyP	*E. coli*	2.17	157.6	0.004	formate	0.065	[4]
rGlyP	*E. coli*	3.3	6.0	0.116	formate	0.09	[5]
STC	*E. coli*	1.7	10.0	0.069	formate	0.105	[6]
CBB	*E. coli*	2.8	18.0	0.039	formate	0.03	[7]
CBB	*K. phaffii*	-	38.5	0.018	methanol	0.25	[8]
rGlyP	*E. coli*	4.2	55.0	0.013	methanol	0.6	[2]
RuMP	*E. coli**	1.2	8.5	0.082	methanol	0.4	[9]
RuMP	*E. coli**	0.96	8.0	0.087	methanol	0.5	[10]
XuMP	*S. cerevisiae*	-	-		methanol	-	[11]
RuMP	*E. coli***	12.5	3.5	0.198	methanol	0.4	[12]
RuMP	*E. coli**	2	4.3	0.161	methanol	0.5	[13]
rGlyP	*K. phaffi*	2.59	47	0.015	methanol	0.156	[14]

*estimated from graph, assuming 0.4 gCDW/L per OD as in Gleizer 2019
**assuming 400 mM methanol was used for cultivation

Supplementary Table 5 | A complete overview of strains used in this study. Plasmid-based (p) or genomic (g) expression of the genes 5,10-methylene-THF dehydrogenase (*mtdA*, UniProt: P55818), 5,10-methenyl-THF cyclohydrolase (*fchA*, UniProt: Q49135) and formate-THF ligase (*fttL*, UniProt: Q83WS0) from *Methylorubrum extorquens AM1*, *gcvTHP*, *sdaA* and *glyA* from *C. necator* under the control of different promoters.

Strain	Genotype	Source
***E. coli* DH5α**	F− λ− Φ80*lac*ZΔM15 Δ(*lac*ZYA-*arg*F)U169 *deo*R *rec*A1 *end*A1 *hsd*R17(rK− mK+) *pho*A *sup*E44 *thi*-1 *gyr*A96 *rel*A1	Lab stock
***E. coli* ST18**	*pro thi hsdR*+ Tpr Smr; chromosome::RP4-2 Tc::Mu-Kan::Tn7/λpir Δ*hem*A	Lab stock
C. necator H16	wild-type	Lab collection
**Δ*phaC1***	*C. necator H16* Δ*phaC1*	Lab collection
**ΔCBB**	Δ*phaC1*, Δ*cbbSL2p*	Claassens 2020

**CRG4**	Δ*CBB* +pSEVA221-P$_{14}$-*mtdA-fch-ftfL* +gP$_{3mut2}$-*gcvTHP* +pSEVA331-P$_{phaC1}$-*sdaA-glyA*	Claassens 2020
**CRG4.5**	Δ*CBB* +gP$_{3mut2}$-*gcvTHP* +pSEVA331-P$_{phaC1}$-*sdaA-glyA*	This study
**CRG5**	Δ*CBB* +gP$_{14}$-*mtdA-fch-ftfL*-Tn5 +gP$_{3mut2}$-*gcvTHP* +pSEVA331-P$_{phaC1}$-*sdaA-glyA*	This study
**CRG5-P$_{Phac1}$-C1**	Δ*CBB* +gP$_{PhaC1}$-*mtdA-fch-ftfL*-Tn5 +gP$_{3mut2}$-*gcvTHP* +pSEVA331-P$_{phaC1}$-*sdaA-glyA*	This study
**CRG5-P$_{3}$-C1**	Δ*CBB* +gP$_{3}$-*mtdA-fch-ftfL*-Tn5 +gP$_{3mut2}$-*gcvTHP* +pSEVA331-P$_{phaC1}$-*sdaA-glyA*	This study
**CRG5-P$_{4}$-C1**	Δ*CBB* +gP$_{4}$-*mtdA-fch-ftfL*-Tn5 +gP$_{3mut2}$-*gcvTHP* +pSEVA331-P$_{phaC1}$-*sdaA-glyA*	This study
**CRG5-P$_{2}$-C1**	Δ*CBB* +gP$_{2}$-*mtdA-fch-ftfL*-Tn5 +gP$_{3mut2}$-*gcvTHP* +pSEVA331-P$_{phaC1}$-*sdaA-glyA*	This study
**CRG5-P$_{14}$-C1-2**	Δ*CBB* +gP$_{14}$-*mtdA-fch-ftfL*-Tn5 +gP$_{3mut2}$-*gcvTHP* +pSEVA331-P$_{phaC1}$-*sdaA-glyA*	This study
**CRG5-P$_{Phac1}$-C1-2**	Δ*CBB* +gP$_{PhaC1}$-*mtdA-fch-ftfL*-Tn5 +gP$_{3mut2}$-*gcvTHP* +pSEVA331-P$_{phaC1}$-*sdaA-glyA*	This study
**CRG5-P$_{3}$-C1-2**	Δ*CBB* +gP$_{3}$-*mtdA-fch-ftfL*-Tn5 +gP$_{3mut2}$-*gcvTHP* +pSEVA331-P$_{phaC1}$-*sdaA-glyA*	This study
**CRG5-P$_{4}$-C1-2**	Δ*CBB* +gP$_{4}$-*mtdA-fch-ftfL*-Tn5 +gP$_{3mut2}$-*gcvTHP* +pSEVA331-P$_{phaC1}$-*sdaA-glyA*	This study
**CRG5-P$_{2}$-C1-2**	Δ*CBB* +gP$_{2}$-*mtdA-fch-ftfL*-Tn5 +gP$_{3mut2}$-*gcvTHP* +pSEVA331-P$_{phaC1}$-*sdaA-glyA*	This study
**CRG5.5**	Δ*CBB* +gP$_{14}$-*mtdA-fch-ftfL*-Tn5 +gP$_{3mut2}$-*gcvTHP*	This study
**CRG6**	Δ*CBB* +gP$_{14}$-*mtdA-fch-ftfL*-Tn5 +gP$_{3mut2}$-*gcvTHP* +gP$_{3}$-*sdaA-glyA*-Tn5	This study
**CRG6-P$_{cat}$-C3**	Δ*CBB* +gP$_{14}$-*mtdA-fch-ftfL*-Tn5 +gP$_{3mut2}$-*gcvTHP* +gP$_{cat}$-*sdaA-glyA*-Tn5	This study
**CRG6-P$_{PhaC1}$-C3**	Δ*CBB* +gP$_{14}$-*mtdA-fch-ftfL*-Tn5 +gP$_{3mut2}$-*gcvTHP* +gP$_{PhaC1}$-*sdaA-glyA*-Tn5	This study
**CRG6-P$_{4}$-C3**	Δ*CBB* +gP$_{14}$-*mtdA-fch-ftfL*-Tn5 +gP$_{3mut2}$-*gcvTHP* +gP$_{4}$-*sdaA-glyA*-Tn5	This study
**CRG6-P$_{3}$-C3-2**	Δ*CBB* +gP$_{14}$-*mtdA-fch-ftfL*-Tn5 +gP$_{3mut2}$-*gcvTHP* +gP$_{3}$-*sdaA-glyA*-Tn5	This study
**CRG6-P$_{cat}$-C3-2**	Δ*CBB* +gP$_{14}$-*mtdA-fch-ftfL*-Tn5 +gP$_{3mut2}$-*gcvTHP* +gP$_{cat}$-*sdaA-glyA*-Tn5	This study
**CRG6-P$_{PhaC1}$-C3-2**	Δ*CBB* +gP$_{14}$-*mtdA-fch-ftfL*-Tn5 +gP$_{3mut2}$-*gcvTHP* +gP$_{PhaC1}$-*sdaA-glyA*-Tn5	This study
**CRG6-P$_{4}$-C3-2**	Δ*CBB* +gP$_{14}$-*mtdA-fch-ftfL*-Tn5 +gP$_{3mut2}$-*gcvTHP* +gP$_{4}$-*sdaA-glyA*-Tn5	This study
**CRG6 Δ*dadA6***	Δ*CBB,* Δ*dadA6* +gP$_{14}$-*mtdA-fch-ftfL*-Tn5 +gP$_{3mut2}$-*gcvTHP* +gP$_{3}$-*sdaA-glyA*-Tn5	This study

Supplementary Table 6 | A complete overview of plasmids used in this study

**Abbreviation**	**Name**	**Source**
**pC1**	pSEVA221-P$_{14}$-*mtdA-fch-ftfL*	Claassens 2020
**pC3**	pSEVA331-P$_{phaC1}$-*sdaA-glyA*	Claassens 2020
**pBAMD-P$_{14}$-C1**	pBAMD1-4-P$_{14}$-*mtdA-fch-ftfL*	This study
**pBAMD-P$_{PhaC1}$-C1**	pBAMD1-4-P$_{PhaC1}$-*mtdA-fch-ftfL*	This study
**pBAMD-P$_{3}$-C1**	pBAMD1-4-P$_{3}$-*mtdA-fch-ftfL*	This study

**pBAMD-P_4-C1**	pBAMD1-4-P_4-*mtdA-fch-ftfL*	This study
**pBAMD-P_2-C1**	pBAMD1-4-P_2-*mtdA-fch-ftfL*	This study
**pBAMD-P_{cat}-C3**	pBAMD1-4-P_{cat}-*sdaA-glyA*	This study
**pBAMD-P_{PhaC1}-C3**	pBAMD1-4-P_{phaC1}-*sdaA-glyA*	This study
**pBAMD-P_3-C3**	pBAMD1-4-P_3-*sdaA-glyA*	This study
**pBAMD-P_4-C3**	pBAMD1-4-P_4-*sdaA-glyA*	This study
**pLO3-dadA6**	pLO3-dadA6	Claassens 2020
**pSIBR004-phaP1-1**	pSIBR004-phaP1-spacer-1	This study
**pSIBR004-phaP1-2**	pSIBR004-phaP1-spacer-2	This study

Supplementary Note 1 | Integration and sequencing of various C1-operon integration strains.

The strain CRG4.5 (CRG4 cured of pC1) was, as expected, unable to grow on minimal media supplemented with formate as sole carbon and energy source (Extended Data Fig. 2). Next, we used this strain to randomly integrate the C1-module using transposon integration vectors with promoter variants of different strengths controlling the synthetic C1-operon[3,15]. Following conjugation of the transposon vectors for each of the different promoters, the cell populations were transferred to formate minimal media to select for formatotrophic growth (Extended Data Fig. 3a). Populations were repeatedly inoculated into fresh medium until the growth rate of the cell population ceased to increase (Extended Data Fig. 3a). After 10 passages, isolated fast-growing clones with different promoter combinations showed similar growth rates and were characterized by sequencing (Extended Data Fig. 4a, Supplementary Table 1).

In these analyses, we found one of these clones (from here on CRG5) to contain two separate insertions of the same P_{14}-C1 operon, indicating it may have selected for optimal expression levels by integrating two copies of the C1 operon (Extended Data Fig. 5a, Supplementary Table 1). The first insertion interrupted the gene encoding a methyl-cis-aconitate hydratase (*acnM*) involved in the 2-methylcitrate cycle for growth on propionate[16], which is redundant for formatotrophic growth conditions. The second insertion was found upstream of the gene encoding a phasin protein in *C. necator* (*phaP1*), which is involved in encapsulating the storage polymer polyhydroxybutyrate (PHB). *phaP1* is highly expressed in PHB producing conditions and can make up 5 % of the total protein in *C. necator*[17]. However, the *C. necator* production platform strains used in this study are incapable of PHB formation due to a *phaC1* knockout, making the *phaP1* expression redundant and possibly making it a good locus for heterologous expression as the C1 operon may benefit from additional expression by read-through from the native *phaP1* promoter.

Sequencing of the CRG5 strains with other promoter variants than P_{14} showed more insertions in the *phaP1* locus. Clones with the C1-module under expression of the promoters P_3, P_4 and P_{14} (2nd clone) all had the operon inserted in the *phaP1* locus 98 nt upstream of the start codon, further highlighting the relevance of this locus (Supplementary Table 3). Overall, the sequencing results from different clones indicate that the weaker promoters (such as P_{14}) were integrated in this high expression locus to support fast formatotrophic growth (Supplementary Table 3). The C1 operon under control of the strongest tested promoter (P_2) however inserted the transposon in the *H16_A3661* (*H16_B0502*) locus but also deleted 189 nt of the *phaP1* promoter region (+123 nt) (Supplementary Table 3). Also, this was the only sequenced clone where the C1 operon inserted anti-sense with respect to the surrounding ORF or promoter directionality. This indicated that expression from the strong P_2 promoter did not require additional expression via read-through from the *phaP1* promoter. The P_{PhaC1}-C1 transposon

integrated at position 590 nt in the short chain dehydrogenase gene *H16_A0258*, possibly associated with PHB binding[18], and a mutation locus also later found in CRG6. The strain with the weakest constitutive promoter (P_{14}) and two C1 operon insertions was further characterized in detail (referred to as CRG5 in the main text).

Supplementary Note 2 | Integration of the C3-operon and the DadA6 route.

To investigate if the growth rate and yield of the rGlyP strains could be further optimized, we next aimed at integrating module 3 using the workflow described above (Extended Data Fig. 1). We cured the CRG5 strain of the last remaining plasmid (pC3). Notably, the created CRG5.5 strain was still able to grow on formate, albeit at a much slower growth rate and optical density (Extended Data Fig. 2). This is in line with our previous study, where we identified glycine oxidase DadA6 as the native glycine assimilation route, which could be used as an alternative to the plasmid-encoded C3-module. The native route wastefully oxidizes glycine to glyoxylate, which is further channeled into 2-phospho-glycerate and central metabolism via the glycerate shunt. The glycine oxidation route is energetically on par with the CBB cycle, limiting the biomass yield of the CRG5.5 strain[3,19].

To re-establish the superior serine route, we transformed CRG5.5 with another set of transposon integration vectors that contained native *C. necator sdaA* and *glyA* genes downstream of another small library of constitutive promoters and selected for formatotrophic growth (Extended Data Fig. 3b). After 10 total passages on formate minimal media, several similarly fast-growing strains (CRG6) were isolated. Among the different constructs, the CRG6 lineage containing the P_3-C3-module seemed to perform best (Extended Data Fig. 4b). Sequencing revealed that in one of the strains the P_3-C3 operon interrupted the *H16_B1976* gene, encoding a putative *tonB*-dependent outer membrane receptor (Extended Data Fig. 5b, Supplementary Table 2). In the other sequenced strain the P_3-C3 operon was inserted into the *prpB* gene located on chromosome 1 (Supplementary Table 3). PrpB, together with AcnM is part of propionate metabolism in *C. necator*[16]. The CRG6 clone with the P_3-C3 operon integrated in the *H16_B1976* gene grew best and was further investigated (referred to as CRG6 further).

To suppress the glycine oxidation route in CRG6 that might compete with the rGlyP, we deleted *dadA6*. However, the doubling time and yield of the CRG6 Δ*dadA6* strain were not significantly altered (Extended Data Fig. 2), indicating that the glycine oxidation pathway did not play a major role for growth of CRG6 on formate.

Supplementary Note 3 | In-depth proteomic analysis of formatotrophic strains.

First, we compared the expression of the rGlyP genes in the CRG4 and CRG6 strains (Fig. 2b, Extended Data Fig. 6a). Here we found the genomic expression of module 1 from strain

CRG6 to have almost reached the expression of the same operon from plasmid in CRG4. As both the plasmid-based and genomic C1 operons are controlled by the same (weak) P_{14} promoter we attribute the consistent high expression from the genome to the duplicate integration in the genome and read-through expression of strong *phaP1* promoter. This also indicates that this module likely requires a relatively high expression for sufficient growth on formate. Expression of module 2 (GCV) remained basically unchanged from CRG4 to CRG6, which was expected, as this module was already genome-integrated previously[3]. The genomic insertion of module 3 of the CRG6 strain however decreased expression compared to the CRG4 strain by over one order of magnitude, possibly indicating that this operon was expressed too strongly from plasmid in the CRG4 strain (Fig. 2b, Extended Data Fig. 6a). Indeed, in the previous study only a narrow range of expression levels were tested for this module.

Next, we investigated the effect of the transposon insertions and mutations on their insertion loci. Expression of genes in the C1-operon integration loci were decreased (*phaP1* decreased 150-fold and *acnM* by a factor of 14) (Extended Data Fig. 6a). The short chain dehydrogenase H16_A0258, which contains a 1 bp deletion in strain CRG6 (Supplementary Table 2) and was also found to be a target of the P_{PhaC1}-C1 transposition event was also downregulated by a factor of 28. The protein encoded by the gene *H16_B1976* and target of the gP_3-C3 transposon insertion was not up or down regulated significantly, possible reflecting the neutrality of this locus in terms of expression and deletion for the cell.

We then compared the proteomes of CRG6 to CRG4 beyond rGlyP and mutation loci (Extended Data Fig. 6a). Interestingly, among the most upregulated proteins in CRG6 compared to CRG4 was 2-amino-3-ketobutyrate CoA ligase (Kbl), which was upregulated by a factor of 26. Kbl, together with threonine dehydrogenase (Tdh) catalyzes the formation of threonine from glycine and acetyl-CoA. Tdh however was not significantly up or down regulated in CRG6, and acetyl-CoA could only be wastefully generated from pyruvate via rGlyP and pyruvate dehydrogenase. This leaves an unclear picture of the relevance of the Kbl upregulation in CRG6. Glutamate dehydrogenase 1 (Gdh1) seems to be slightly upregulated (~4 fold) in CRG6 over CRG4. This however, is due to a 13-fold downregulation of Gdh1 in CRG4 compared to the CBB cycle strain. PHB de-polymerase (PhaZ6), as well as formate dehydrogenase subunits FdhA1 and FdhC are downregulated by ~2-fold, which possibly further relates to the biomass yield gain of CRG6 over CRG4. Overall, proteins associated with drug efflux, *tonB*-dependent receptors, ferric uptake and L-leucine/L-phenylalanine ABC transporters where upregulated in CRG6 relative to CRG4 (Extended Data Fig. 6a).

Next, we used measured protein intensities to allow approximate quantification of the total proteome fractions allocated to different metabolic tasks (Fig. 2c). We found the rGlyP to make up 25 % of the quantified proteome in CRG4 and around 14 % in CRG6. In strains CRG4 and

CRG6 the C1-module accounted for 10 % and 8 % respectively. Expression of module 2 in CRG4 in CRG6 made up 5 %, while module 3 contributed to a fraction of 9 % in CRG4 and only 0.8 % in CRG6. The C1-operon insertion between the *phaP1* promoter and the *phaP1* CDS further led to a decrease of its quantified proteome fraction from ~1 % to ~0.01 % in CRG4 versus CRG6 (see Source data). To check the effect of the *phaP1* deletion alone on formatotrophic growth, we compared the CBB-cycle formatotrophic phenotypes of *C. necator H16* Δ*phaC1* and Δ*phaC1* Δ*phap1* strains. This revealed no significant improvements in growth rate or yield, suggesting that this deletion is not a major factor explaining the faster growth of the CRG5/6 strains (Extended Data Fig. 7). In addition, as expected, in the plasmid-free CRG6 strain plasmid replication and marker protein expression (~0.4 % of quantified proteome in CRG4) was absent, which could save some cellular energy and resource investment and could speed up the growth (Fig. 2c). Another change in the proteome allocation between the CBB cycle strain and the CRG strains was the sharply decreased proteome allocation towards hydrogenases from 2.9% to 0.1% (Fig. 2c). This can be related to the hydrogenase activator knockout mutation (HoxA) observed already during the previous creation of CRG4.

The proteome fraction associated with RuBisCO in the CBB strain made up ~1.9 %, with all of the CBB cycle enzymes together only accounting for 6 % of the proteome. Despite RuBisCOs famously high protein content in higher plants our findings are in line with previous studies of bacterial RuBisCO protein abundance[20]. The RuBisCO protein concentration is also negatively correlated with elevated CO_2 conditions and in our study all strains were grown at high (10 %) CO_2[21]. We find the hydrogen metabolism proteins to make up a fraction of 3 % in the CBB strain. However, the CBB cycle and hydrogen utilization proteins constitute only 0.01 % and 0.1 % respectively of the total quantified proteome in the rGlyP strains (Fig. 2c).
Last, we compared the proteomes of CRG4 and CRG6 to the CBB strain (Extended Data Fig. 6b,c). Here we found several global proteome changes that we could partially relate to mutations already present in the CRG4 lineage. In all rGlyP dependent strains the CBB cycle is downregulated by two orders of magnitude, which was expected due to the deletion of the transcriptional activator *cbbR*[3]. Also, the hydrogen utilization proteome is downregulated by a similar degree in line with the *hoxA* mutation already present in the CRG4 strain lineage. This indicated that the CRG4 and CRG6 strains lost their "readiness"[22] to use hydrogen as substrate, which is indeed redundant for formatotrophic growth. We further found propionyl-CoA transferase Pct to be upregulated by factor of 16, possibly related to mutations found in the 2-methylcitrate cycle genes *acnM* and *prpB*. Since the rGlyP is connected to central metabolism at the level of pyruvate, we expected more gluconeogenic flux compared to the CBB strains. While this was not reflected by significantly altered expression of the genes

encoding PEP synthetase, Pyruvate carboxylase and PEP carboxykinase, we did observe pyruvate kinase 3 to be downregulated by a factor of 16 in both CRG4 and CRG6 (Extended Data Fig. 6b,c).

Supplementary Note 4 | The rGlyP is already energetically optimal in CRG6

Apart from the three rGlyP assimilation modules, another key enzyme for operation of the rGlyP (and the CBB cycle) is the native, energy-generating NAD^+-dependent, soluble formate dehydrogenase (FDH). For growth via the CBB cycle, all formate needs to be oxidized via FDH activity, whereas the rGlyP depends on a balancing of flux between formate assimilation and formate oxidation. In case FDH activity is too high relative to the formate assimilation flux, over-oxidation of formate (and resulting futile energy generation) could decrease the yield. To test this, we gradually inhibited molybdenum-dependent sFDH activity via competitive titration with the non-active metal tungsten, based on previously demonstrated methods[23,24]. However, no further increases in biomass could be obtained over a range of almost 6 orders of magnitude of supplemented tungsten (Extended Data Fig. 8a-e).

Another potential metabolic inefficiency that could potentially limit the yield of the rGlyP would be oxidation of glycine into glyoxylate. In our previous work it was observed that *C. necator* harbors a glycine oxidase (DadA6) that could lead to a wasteful by-pass route for the rGlyP[3]. Hence, we deleted the gene encoding DadA6 in CRG6. However, this CRG6 Δ*dadA6* strain grew similarly fast and to the same biomass density in batch as for CRG6 (Fig. 2a), showing that the glycine oxidase is probably not playing a significant role in the CRG6 rGlyP metabolism. Still, to fully rule out this by-pass route, the CRG6 Δ*dadA6* strain was further used for biomass yield determination in bioreactors.

Supplementary figures

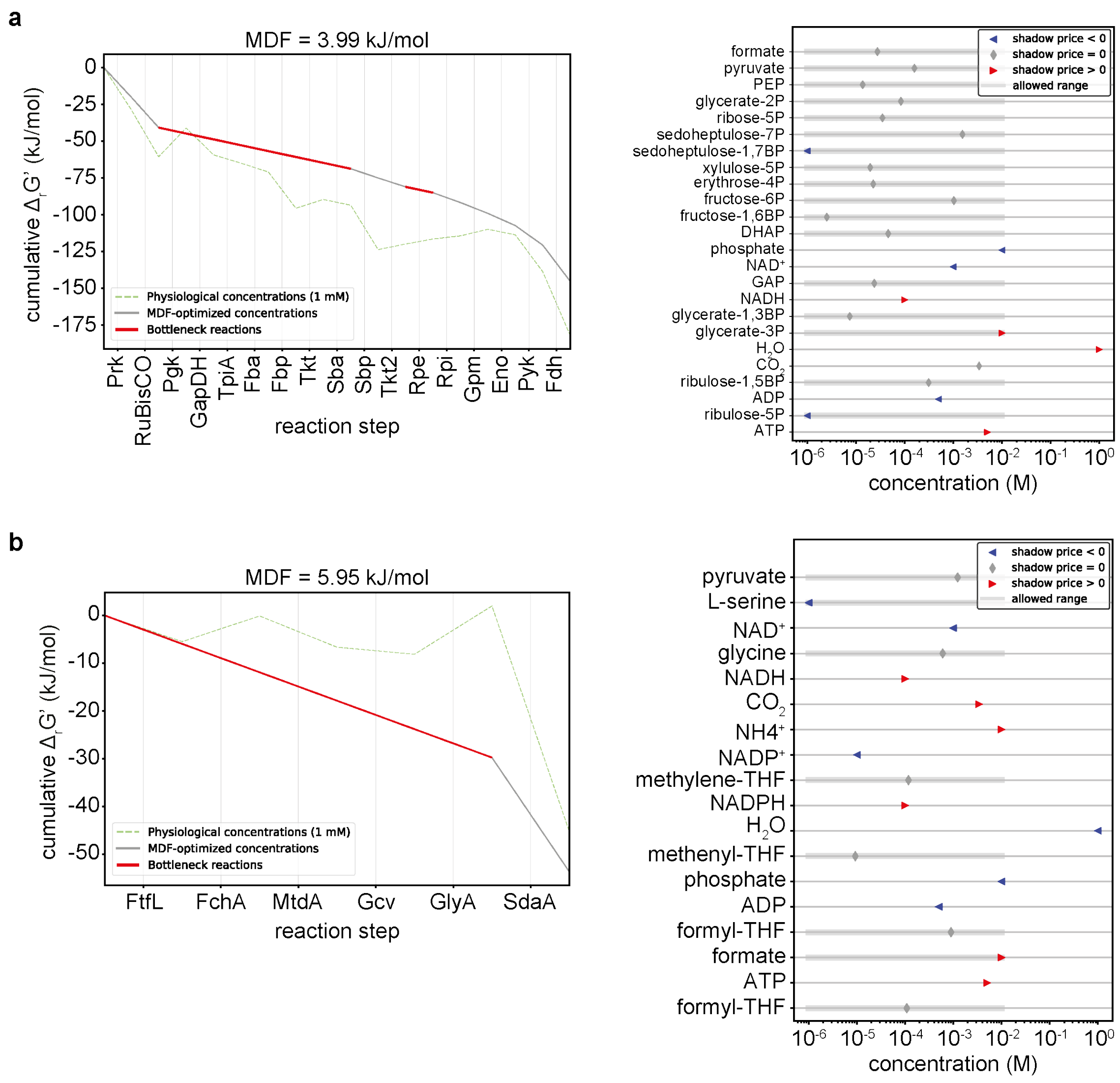

Supplementary Fig, 1: Max-Min driving force (MDF) analysis. MDF was used to compare the overall pathway thermodynamics from formate to pyruvate for the Calvin-Benson-Bassham cycle (**a**) and the reductive glycine pathway (**b**). Left panels show the energetic profile and the right panels the metabolite concentrations after MDF optimization.The green lines show $\Delta_rG'^o$ values of pathway reactions at ph=7.5, the grey lines Δ_rG' values after MDF optimization and the red lines indicate bottleneck reactions.

References

1. Grunwald, S. *et al.* Kinetic and stoichiometric characterization of organoautotrophic growth of *Ralstonia eutropha* on formic acid in fed- batch and continuous cultures. *Microbial Biotechnology* **8**, 155–163 (2015).
2. Kim, S. *et al.* Growth of *E. coli* on formate and methanol via the reductive glycine pathway. *Nature Chemical Biology* **16**, 538–545 (2020).
3. Claassens, N. J. *et al.* Replacing the Calvin cycle with the reductive glycine pathway in *Cupriavidus necator*. *Metabolic Engineering* **62**, 30–41 (2020).
4. Bang, J., Hwang, C. H., Ahn, J. H., Lee, J. A. & Lee, S. Y. *Escherichia coli* is engineered to grow on CO_2 and formic acid. *Nature Microbiology* **5**, 1459–1463 (2020).
5. Kim, S. *et al.* Optimizing *E. coli* as a formatotrophic platform for bioproduction via the reductive glycine pathway. *Front. Bioeng. Biotechnol.* **11**, 1091899 (2023).
6. Wenk, S. *et al.* Evolution-assisted engineering of *E. coli* enables growth on formic acid at ambient CO_2 via the Serine Threonine Cycle. *Metabolic Engineering* **88**, 14–24 (2025).
7. Gleizer, S. *et al.* Conversion of *Escherichia coli* to Generate All Biomass Carbon from CO_2. *Cell* **179**, 1255-1263.e12 (2019).
8. Gassler, T. *et al.* The industrial yeast *Pichia pastoris* is converted from a heterotroph into an autotroph capable of growth on CO_2. *Nature Biotechnology* **38**, 210–216 (2020).
9. Chen, F. Y.-H., Jung, H.-W., Tsuei, C.-Y. & Liao, J. C. Converting *Escherichia coli* to a Synthetic Methylotroph Growing Solely on Methanol. *Cell* **182**, 933-946.e14 (2020).

10. Keller, P. *et al.* Generation of an *Escherichia coli* strain growing on methanol via the ribulose monophosphate cycle. *Nat Commun* **13**, 5243 (2022).

11. Zhan, C. *et al.* Reprogramming methanol utilization pathways to convert *Saccharomyces cerevisiae* to a synthetic methylotroph. *Nat Catal* **6**, 435–450 (2023).

12. Nieh, L.-Y. *et al.* Evolutionary engineering of methylotrophic *E. coli* enables fast growth on methanol. *Nat Commun* **15**, 8840 (2024).

13. Reiter, M. A. *et al.* A synthetic methylotrophic *Escherichia coli* as a chassis for bioproduction from methanol. *Nature Catalysis* **7**, 560–573 (2024).

14. Guo, Y. *et al.* Engineering yeasts to Co-utilize methanol or formate coupled with CO_2 fixation. *Metabolic Engineering* **84**, 1–12 (2024).

15. Mutalik, V. K. *et al.* Precise and reliable gene expression via standard transcription and translation initiation elements. *Nature Methods* **10**, 354–360 (2013).

16. Brämer, C. O. & Steinbüchel, A. The methylcitric acid pathway in *Ralstonia eutropha*: New genes identified involved in propionate metabolism. *Microbiology* **147**, 2203–2214 (2001).

17. Wieczorek, R., Pries, A., Steinbuchel, A. & Mayer, F. Analysis of a 24-kilodalton protein associated with the polyhydroxyalkanoic acid granules in *Alcaligenes eutrophus*. *Journal of Bacteriology* **177**, 2425–2435 (1995).

18. Pfeiffer, D. Identifizierung und Funktionsanalyse neuartiger Proteine des PHB-Granulumkomplexes von *Ralstonia eutropha H16*. (Universität Stuttgart, 2013).

19. Zeng, A. & Claassens, N. J. *One-Carbon Feedstocks for Sustainable Bioproduction*. (2022).

20. Raven, J. A. Rubisco: Still the most abundant protein of Earth? *New Phytologist* **198**, 1–3 (2013).
21. Losh, J. L., Young, J. N. & Morel, F. M. M. Rubisco is a small fraction of total protein in marine phytoplankton. *New Phytologist* **198**, 52–58 (2013).
22. Jahn, M. *et al.* Protein allocation and utilization in the versatile chemolithoautotroph *Cupriavidus necator*. *eLife* **10**, 1–39 (2021).
23. Pinsent, J. The need for selenite and molybdate in the formation of formic dehydrogenase by members of the *Coli-aerogenes* group of bacteria. *Biochemical Journal* **57**, 10–16 (1954).
24. Friedebold, J. & Bowien, B. Physiological and biochemical characterization of the soluble formate dehydrogenase, a molybdoenzyme from *Alcaligenes eutrophus*. *J Bacteriol* **175**, 4719–4728 (1993).
