# Supplementary material for: One-carbon fixation via the synthetic reductive glycine pathway exceeds yield of the Calvin cycle
Source: Nat Microbiol. 2025 Feb 27;10(3):646–53. doi: 10.1038/s41564-025-01941-9 (PMC11879842; doi:10.1038/s41564-025-01941-9)
Supplement: Supplementary file 2 — Reporting Summary [file 41564_2025_1941_MOESM2_ESM.pdf]

nature portfolio

Corresponding author(s): Beau Dronsella
Nico Claassens

Last updated by author(s): Jan 10, 2025

Reporting Summary

Nature Portfolio wishes to improve the reproducibility of the work that we publish. This form provides structure for consistency and transparency in reporting. For further information on Nature Portfolio policies, see our Editorial Policies and the Editorial Policy Checklist.

Statistics

For all statistical analyses, confirm that the following items are present in the figure legend, table legend, main text, or Methods section.

n/a	Confirmed	
☐	☒	The exact sample size (*n*) for each experimental group/condition, given as a discrete number and unit of measurement
☐	☒	A statement on whether measurements were taken from distinct samples or whether the same sample was measured repeatedly
☐	☒	The statistical test(s) used AND whether they are one- or two-sided<br>*Only common tests should be described solely by name; describe more complex techniques in the Methods section.*
☒	☐	A description of all covariates tested
☒	☐	A description of any assumptions or corrections, such as tests of normality and adjustment for multiple comparisons
☐	☒	A full description of the statistical parameters including central tendency (e.g. means) or other basic estimates (e.g. regression coefficient) AND variation (e.g. standard deviation) or associated estimates of uncertainty (e.g. confidence intervals)
☐	☒	For null hypothesis testing, the test statistic (e.g. *F*, *t*, *r*) with confidence intervals, effect sizes, degrees of freedom and *P* value noted<br>*Give P values as exact values whenever suitable.*
☒	☐	For Bayesian analysis, information on the choice of priors and Markov chain Monte Carlo settings
☒	☐	For hierarchical and complex designs, identification of the appropriate level for tests and full reporting of outcomes
☒	☐	Estimates of effect sizes (e.g. Cohen's *d*, Pearson's *r*), indicating how they were calculated

Our web collection on statistics for biologists contains articles on many of the points above.

Software and code

Policy information about availability of computer code

Data collection	No special software was used.
Data analysis	The following software was used: MatLab R2019a, equilibrator-api 0.2.5, Geneious 8.1, Breseq (v0.36.1), SnapGene 8.0.1, Graph Pad Prism 10.1.0, Adobe Illustrator 2020, Perseus 1.5.1.6

For manuscripts utilizing custom algorithms or software that are central to the research but not yet described in published literature, software must be made available to editors and reviewers. We strongly encourage code deposition in a community repository (e.g. GitHub). See the Nature Portfolio guidelines for submitting code & software for further information.

Data

Policy information about availability of data

All manuscripts must include a data availability statement. This statement should provide the following information, where applicable:

- Accession codes, unique identifiers, or web links for publicly available datasets
- A description of any restrictions on data availability
- For clinical datasets or third party data, please ensure that the statement adheres to our policy

Data supporting the findings of this study are found in the paper, the extended data figures, the supplementary information, or the source data files. Sequence data of plasmids constructed and/or used in this study was made available on EDMOND (https://doi.org/10.17617/3.FSBOQE). Whole-genome sequencing data were deposited on the NCBI sequencing read archive (SRA) under the BioProject number PRJNA1209586. The mass spectrometry proteomics data have been deposited

to the ProteomeXchange Consortium via the PRIDE partner repository with the dataset identifier PXD059545. Further information, bacterial strains and materials related to this work can be obtained from the corresponding authors. The GEM of C. necator -RehMBEL1391_sbml_L3V1, the code and the associated results obtained in this study have been deposited in the following GitHub repository: https://github.com/benit005/Formate_assimilation_rGlyPvsCBB_Cupriavidus_necator

Research involving human participants, their data, or biological material

Policy information about studies with human participants or human data. See also policy information about sex, gender (identity/presentation), and sexual orientation and race, ethnicity and racism.

Reporting on sex and gender	N/A
Reporting on race, ethnicity, or other socially relevant groupings	N/A
Population characteristics	N/A
Recruitment	N/A
Ethics oversight	N/A

Note that full information on the approval of the study protocol must also be provided in the manuscript.

Field-specific reporting

Please select the one below that is the best fit for your research. If you are not sure, read the appropriate sections before making your selection.

☒ Life sciences ☐ Behavioural & social sciences ☐ Ecological, evolutionary & environmental sciences

For a reference copy of the document with all sections, see nature.com/documents/nr-reporting-summary-flat.pdf

Life sciences study design

All studies must disclose on these points even when the disclosure is negative.

Sample size	Sample sizes for proteomics were in biological triplicates, which is a generally accepted number for similar microbial experiments. Sample size of growth experiments were 2-3 technical replicates with the experiments being repeated at least 3 times to ensure reproducibility, which reflects the state of the art in the field (10.1038/s41467-024-53762-9). The chemostat biomass yield experiment was conducted in the same way as an established reference in the field (10.1111/1751-7915.12149), making results more comparable. We harvested over the course of 3 days a total of 9 samples to assure technical replication. We further were able to benchmark our data to the aforementioned reference, have several OD600 based growth experiments showing similar trends in biomass formation, and have preprinted flask yield data further confirming our results.
Data exclusions	No data were excluded.
Replication	Growth experiments in glass tubes and plate readers were repeated 3 times to ensure reproducibility. Biomass yields are derived from 9 technical replicates taken from the chemostat in steady-state over the course of 3 days.
Randomization	Biological replicates were chosen at random from plate, otherwise no further randomization was performed.
Blinding	Blinding was not performed, as this study was conducted using a single microorganism in a controlled laboratory environment, with appropriate controls to avoid bias.

Reporting for specific materials, systems and methods

We require information from authors about some types of materials, experimental systems and methods used in many studies. Here, indicate whether each material, system or method listed is relevant to your study. If you are not sure if a list item applies to your research, read the appropriate section before selecting a response.

Materials & experimental systems

n/a	Involved in the study
☒	☐ Antibodies
☒	☐ Eukaryotic cell lines
☒	☐ Palaeontology and archaeology
☒	☐ Animals and other organisms
☒	☐ Clinical data
☒	☐ Dual use research of concern
☒	☐ Plants

Methods

n/a	Involved in the study
☒	☐ ChIP-seq
☒	☐ Flow cytometry
☒	☐ MRI-based neuroimaging

Plants

Seed stocks	N/A
Novel plant genotypes	N/A
Authentication	N/A
